# MMP-7 as a non-invasive biomarker in interstitial lung diseases and other chronic respiratory diseases: a narrative review

**DOI:** 10.3389/fmed.2026.1930150

**Published:** 2026-09-28

**Authors:** Marian Marius Padureanu, Ileana Marinescu, Denisa Maria Mitroi, Radu Mihai Coanda, Simona Maria Rosu, Ana Stefania Stoica, Mihnea Marian Pomacu, Ionelia Sorina Stan, Ionela Alina Croitoru, Simona Banicioiu, Anca Rosu, Viorel Biciusca

**Affiliations:** 1 Doctoral School, University of Medicine and Pharmacy, Craiova, Romania; 2 Department of Psychiatry, Faculty of Medicine, University of Medicine and Pharmacy of Craiova, Craiova, Romania; 3 Psychiatry Clinic, Craiova Clinical Hospital for Neuropsychiatry, Craiova, Romania; 4 Department of Internal Medicine, Faculty of Medicine, University of Medicine and Pharmacy of Craiova, Craiova, Romania; 5 Department of Pneumology, Faculty of Medicine, University of Medicine and Pharmacy Carol Davila of Bucharest, Bucharest, Romania; 6 Rheumatology Research Center, University of Medicine and Pharmacy of Craiova, Craiova, Romania

**Keywords:** biomarker, chronic respiratory diseases, diagnostic, interstitial lung disease, MMP-7, prognostic, progressive pulmonary fibrosis

## Abstract

**Background:**

Interstitial lung diseases (ILDs) are a heterogeneous group of disorders in which epithelial injury, inflammation and fibrosis lead to progressive loss of lung function. Several ILDs other than idiopathic pulmonary fibrosis (IPF), including connective tissue disease-associated ILD (CTD-ILD), fibrotic hypersensitivity pneumonitis and fibrotic nonspecific interstitial pneumonia, may develop a progressive fibrosing phenotype, and distinguishing fibrotic from non-fibrotic disease is clinically important. Matrix metalloproteinase-7 (MMP-7), a circulating biomarker of extracellular matrix remodeling, epithelial injury and inflammation, has been investigated as a non-invasive marker of fibrosing lung disease, but the evidence remains fragmented and unsynthesized.

**Aim:**

To summarize current evidence on the diagnostic and prognostic value of MMP-7 in interstitial lung diseases and other chronic respiratory diseases, with particular emphasis on fibrosing lung disorders.

**Materials and methods:**

A narrative review was performed using PubMed/MEDLINE, Scopus and Web of Science. Search terms combined “MMP-7” and “matrix metalloproteinase-7” with terms for interstitial lung disease, CTD-ILD, fibrotic lung diseases, sarcoidosis, hypersensitivity pneumonitis, progressive pulmonary fibrosis, organizing pneumonia and chronic respiratory diseases. Studies were selected on their evaluation of the diagnostic, prognostic or monitoring role of MMP-7.

**Results and discussion:**

In IPF, MMP-7 shows strong diagnostic and prognostic performance and is associated with disease severity, functional decline and mortality. Elevated levels are consistently reported in CTD-ILD, particularly systemic sclerosis- and rheumatoid arthritis-associated ILD, where they correlate with impaired pulmonary function, radiological fibrosis and disease activity. Increased concentrations have also been described in fibrotic hypersensitivity pneumonitis, fibrotic nonspecific interstitial pneumonia and organizing pneumonia, with conflicting findings in sarcoidosis and chronic obstructive pulmonary disease and limited or predominantly experimental data in asthma and bronchiectasis, where elevation reflects epithelial injury and airway remodeling rather than disease-specific pathology. Across these disorders MMP-7 mirrors shared pathways of epithelial damage and matrix turnover, supporting its potential utility in diagnosis and risk stratification, while its value for monitoring disease activity or treatment response remains unproven.

**Conclusion:**

MMP-7 is best regarded as a marker of active fibrotic remodeling rather than of any single disease, with its clearest value in fibrosing ILD and within multimarker strategies rather than as a standalone test.

## 1 Introduction

Interstitial lung diseases (ILDs) comprise a heterogeneous group of disorders characterized by varying degrees of inflammation, epithelial injury, and pulmonary fibrosis, and contribute substantially to respiratory morbidity and mortality worldwide (1). Although fibrosis is a hallmark of idiopathic pulmonary fibrosis (IPF), it is not exclusive to this condition: connective tissue disease-associated ILD (CTD-ILD), chronic hypersensitivity pneumonitis (HP), fibrotic nonspecific interstitial pneumonia (NSIP), sarcoidosis, and organizing pneumonia may all develop a progressive fibrosing phenotype characterized by irreversible extracellular matrix deposition, declining lung function, and increased mortality. Other ILDs remain predominantly inflammatory and may respond to immunomodulatory treatment without developing extensive fibrosis. Distinguishing fibrotic from non-fibrotic disease is therefore of considerable clinical importance, as it influences prognosis, therapeutic decision-making, and monitoring.

Current diagnostic assessment relies principally on high-resolution computed tomography (HRCT), pulmonary function testing (PFT) and, in selected cases, surgical or transbronchial lung biopsy. These modalities identify structural abnormality that is already established, carry interobserver variability, and, in the case of biopsy, involve procedural risk that is not always acceptable in patients with impaired lung function. They are therefore better suited to confirming disease than to detecting it early or to tracking its activity over short intervals.

This has driven interest in circulating biomarkers capable of reflecting fibrotic remodeling before irreversible damage becomes apparent. An ideal candidate would be measurable in peripheral blood, would reflect the biological processes driving fibrosis rather than their structural consequences, and would be suitable for repeated measurement during follow-up. Matrix metalloproteinase-7 (MMP-7) is a secreted proteolytic enzyme involved in extracellular matrix turnover, epithelial repair, and immune signaling, and is among the most extensively investigated of these candidates. Elevated circulating concentrations have been reported across fibrosing ILDs—most consistently in IPF, where they correlate with disease severity, functional decline, and progression, and in CTD-ILD, where they are associated with pulmonary involvement and impaired lung function (2–5). MMP-7 has also been examined in chronic obstructive pulmonary disease (COPD), asthma, and bronchiectasis, although the evidence in these conditions is considerably less mature (6).

Despite this interest, the available evidence remains fragmented across diseases, populations, biological specimens, and clinical endpoints, and no synthesis has critically evaluated MMP-7 across the full range of conditions in which it has been studied. This narrative review therefore summarizes the biological functions of MMP-7 and evaluates current evidence for its diagnostic and prognostic value, first in interstitial lung diseases—IPF, CTD-ILD, HP, fibrotic NSIP, sarcoidosis, organizing pneumonia, and progressive pulmonary fibrosis as a cross-cutting phenotype—and subsequently in COPD, asthma, and bronchiectasis.

## 2 Materials and methods

A comprehensive literature search was conducted using the PubMed/MEDLINE, Scopus, and Web of Science databases, supplemented by manual screening of reference lists from relevant publications. Literature searches were conducted using combinations of the following terms: “MMP-7”, “matrix metalloproteinase-7”, “matrilysin”, “idiopathic pulmonary fibrosis”, “connective tissue disease-associated interstitial lung disease”, “CTD-ILD”, “systemic sclerosis-associated interstitial lung disease”, “rheumatoid arthritis-associated interstitial lung disease”, “mixed connective tissue disease”, “nonspecific interstitial pneumonia”, “organizing pneumonia”, “cryptogenic organizing pneumonia”, “progressive pulmonary fibrosis”, “sarcoidosis”, “hypersensitivity pneumonitis”, “chronic obstructive pulmonary disease”, “asthma”, “bronchiectasis”, “biomarker”, “diagnosis”, and “prognosis”. The search aimed to identify relevant publications available up to March 2026.

The review is organized into two main sections: interstitial lung diseases, including IPF, CTD-ILD, NSIP, HP, sarcoidosis, the cross-cutting progressive pulmonary fibrosis (PPF) phenotype, and organizing pneumonia (OP); and other chronic respiratory diseases, including COPD, asthma, and bronchiectasis.

Conditions were selected according to two explicit criteria: (I) a shared pathobiology involving epithelial injury, aberrant repair, and extracellular matrix remodeling, the processes in which MMP-7 has been implicated; and (II) the availability of at least one clinical study reporting MMP-7 levels in human subjects with that condition. Conditions meeting the first criterion but not the second—including pulmonary alveolar proteinosis, lymphangioleiomyomatosis, pneumoconioses, and drug-induced ILD—were not addressed as separate sections, since no primary clinical data on MMP-7 were identified for them. Cystic fibrosis was excluded as a distinct entity, although bronchiectasis arising in that context is discussed within the bronchiectasis section. This selection reflects the current distribution of available evidence rather than the full spectrum of chronic respiratory diseases and therefore represents a scope limitation of the present review.

Original research articles, observational studies, clinical trials, systematic reviews, meta-analyses, and relevant clinical practice guidelines published in English were considered for inclusion. Particular emphasis was placed on studies evaluating circulating, airway, or tissue levels of MMP-7 and their associations with clinically relevant outcomes, including disease presence, radiological severity, pulmonary function impairment, disease progression, exacerbation frequency, hospitalization, transplant-free survival, and mortality.

Articles without direct relevance to MMP-7, experimental studies lacking clear translational or clinical implications, conference abstracts with insufficient methodological information, and duplicate publications were excluded. When multiple reports from the same patient cohort were identified, priority was given to the most comprehensive or most informative publication.

Given the heterogeneity of study populations, disease phenotypes, biological specimens, analytical methodologies, and reported outcomes, findings were synthesized qualitatively rather than through formal meta-analysis. The review weighs the consistency of evidence across studies and considers the translational relevance of MMP-7 as a diagnostic and prognostic biomarker in chronic respiratory diseases, particularly fibrosing interstitial lung diseases.

## 3 General notions about MMP-7

Matrix metalloproteinases (MMPs) are a family of zinc-dependent endopeptidases that degrade extracellular matrix (ECM) components and participate in tissue remodeling and repair. Dysregulated MMP activity contributes to pathological matrix remodeling and excessive matrix deposition in fibrotic disorders (7, 8).

### 3.1 Structure and activation

MMP-7, also known as matrilysin-1, is one of the smallest members of the MMP family and exhibits a distinct structure. It is synthesized as a latent zymogen, pro-MMP-7, consisting of a propeptide that maintains the enzyme in an inactive state and a catalytic domain containing the zinc-binding site essential for proteolytic activity. Activation involves proteolytic removal of the propeptide and can be mediated by proteases such as plasmin and other MMPs, or occur via autocatalytic mechanisms under favorable microenvironmental conditions (8–10).

Unlike most MMPs, MMP-7 lacks the C-terminal hemopexin-like domain. This structural feature contributes to its accessibility to a wide range of substrates and to its distinct proteolytic profile. Upon activation, MMP-7 can cleave numerous extracellular matrix components, including elastin, gelatin, fibronectin, laminin, and proteoglycans (11, 12).

### 3.2 Biological functions

MMP-7 participates in extracellular matrix remodeling and in the regulation of interactions between epithelial cells and the tissue microenvironment. Its broad substrate spectrum allows it to modulate both structural matrix components and cell-surface proteins, thereby contributing to cell migration, tissue repair, and epithelial remodeling (7, 13, 14).

In the lung, MMP-7 is predominantly produced by injured alveolar type II and bronchial epithelial cells, with activated macrophages contributing further; fibroblasts, neutrophils and other inflammatory cells contribute only under certain conditions (Figure 1). Following epithelial injury, inflammatory mediators such as tumor necrosis factor-alpha (TNF-α), interleukin-1 beta (IL-1β) and interleukin-13 (IL-13), released by macrophages and T lymphocytes, upregulate MMP-7 expression in epithelial cells. One function particularly relevant to fibrotic lung disease is the cleavage of E-cadherin, a protein essential for maintaining epithelial intercellular junctions; disruption of these junctions promotes loss of epithelial barrier integrity and alterations in cellular phenotype. Through the release of matrix-sequestered growth factors and chemotactic mediators, MMP-7 also influences the recruitment and activation of fibroblasts, macrophages and other immune cells (15–17).

**Figure 1:**
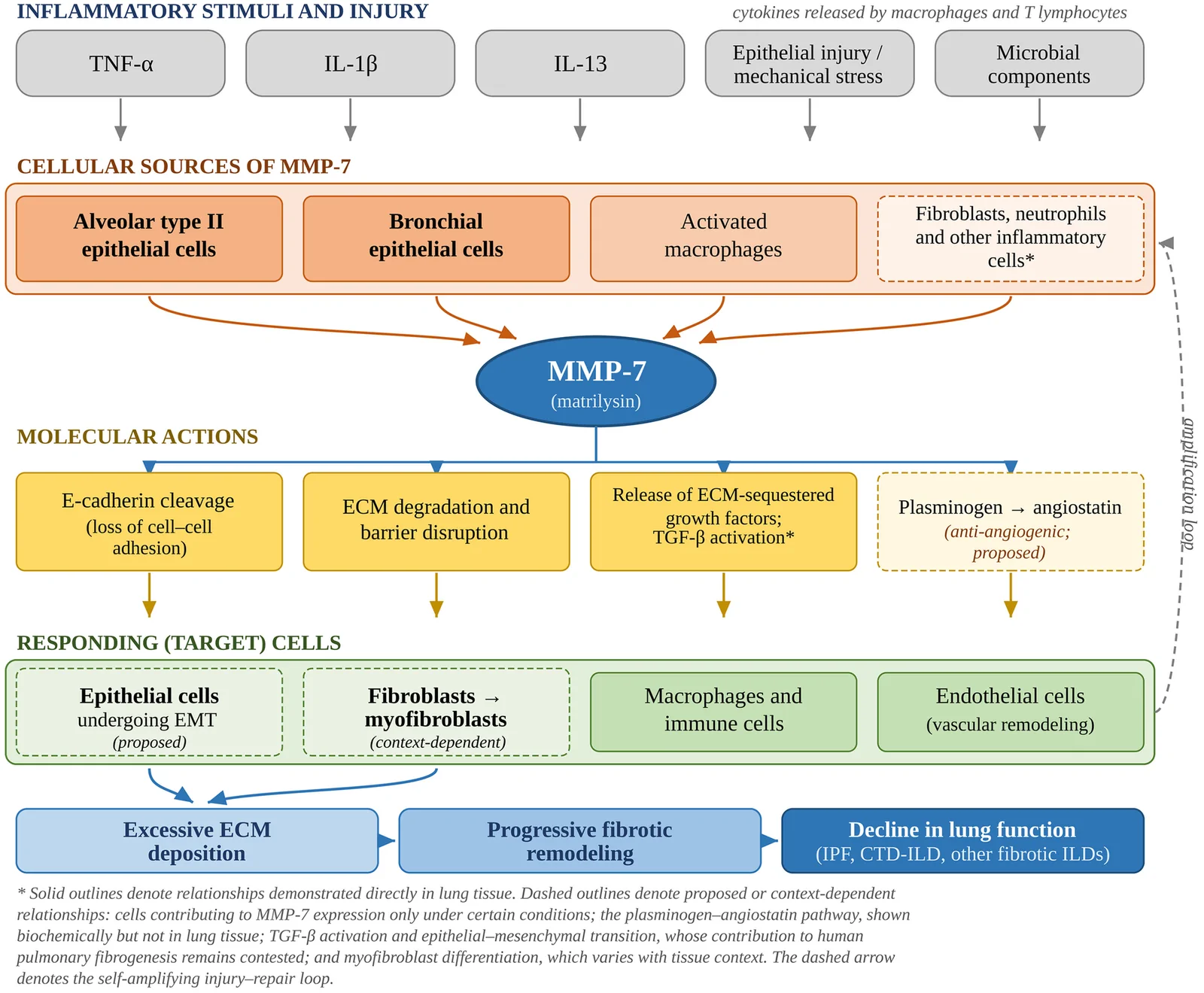
Cellular sources and targets of MMP-7 in the pathogenesis of pulmonary fibrosis. Injured alveolar type II and bronchial epithelial cells are the principal sources of MMP-7, with activated macrophages contributing further; fibroblasts, neutrophils and other inflammatory cells contribute only under certain conditions. Secretion is induced by pro-inflammatory mediators (TNF-α, IL-1β, IL-13) released by macrophages and T lymphocytes, and by epithelial injury, mechanical stress and microbial components. Once secreted, MMP-7 cleaves E-cadherin with loss of cell–cell adhesion, degrades extracellular matrix components and disrupts the epithelial barrier, liberates matrix-sequestered growth factors and activates transforming growth factor beta (TGF-β), and—in a pathway demonstrated biochemically but not yet confirmed in lung tissue—converts plasminogen to angiostatin. These actions are exerted on epithelial cells, in which epithelial–mesenchymal transition has been proposed but remains contested in human pulmonary fibrogenesis; on fibroblasts, in which myofibroblast differentiation is context-dependent; on macrophages and other immune cells; and on endothelial cells, influencing vascular remodeling. The resulting excessive extracellular matrix deposition and progressive fibrotic remodeling culminate in decline in lung function. Solid outlines denote relationships demonstrated directly in lung tissue; dashed outlines denote proposed or context-dependent relationships. The dashed arrow denotes the self-amplifying injury–repair loop. TNF-α, tumor necrosis factor-alpha; IL-1β, interleukin-1 beta; IL-13, interleukin-13; MMP-7, matrix metalloproteinase-7; ECM, extracellular matrix; EMT, epithelial–mesenchymal transition; TGF-β, transforming growth factor beta; IPF, idiopathic pulmonary fibrosis; CTD-ILD, connective tissue disease-associated interstitial lung disease; ILD, interstitial lung disease. Original figure created by the authors.

The involvement of MMP-7 in epithelial–mesenchymal transition (EMT) has been proposed on the basis of experimental observations regarding the loss of epithelial markers and altered cellular behavior in the presence of the enzyme's proteolytic activity. The precise contribution of EMT to human pulmonary fibrogenesis nevertheless remains controversial, and the role of MMP-7 must be interpreted within the broader context of epithelial plasticity and epithelium–mesenchyme interactions (16–18).

MMP-7 can also influence the availability of extracellular mediators through cleavage of matrix proteins and cell-surface molecules, a process that in certain experimental contexts generates proteolytic fragments with distinct biological activity. One example is the processing of plasminogen to form angiostatin, an endogenous inhibitor of angiogenesis (19). This activity has been demonstrated *in vitro* rather than in lung tissue, and whether it operates at physiologically meaningful levels in the fibrotic lung is unknown; it is presented here as a proposed mechanism.

MMP-7 should therefore be regarded as an enzyme with pleiotropic functions, situated at the interface of extracellular matrix remodeling, epithelial injury, inflammation and tissue repair, whose activity may have differing consequences depending on the tissue microenvironment and disease context (7, 13, 14, 20, 21).

### 3.3 Expression and regulation

MMP-7 expression is regulated at the transcriptional level and can be induced by inflammatory stimuli and by alterations in the tissue microenvironment. In injured lung tissue, cytokines such as TNF-α and IL-1β, together with other inflammatory mediators, contribute to increased MMP-7 expression in epithelial cells; microbial components and mechanical stress may also participate depending on the biological context (Figure 2) (9, 16, 17).

**Figure 2:**
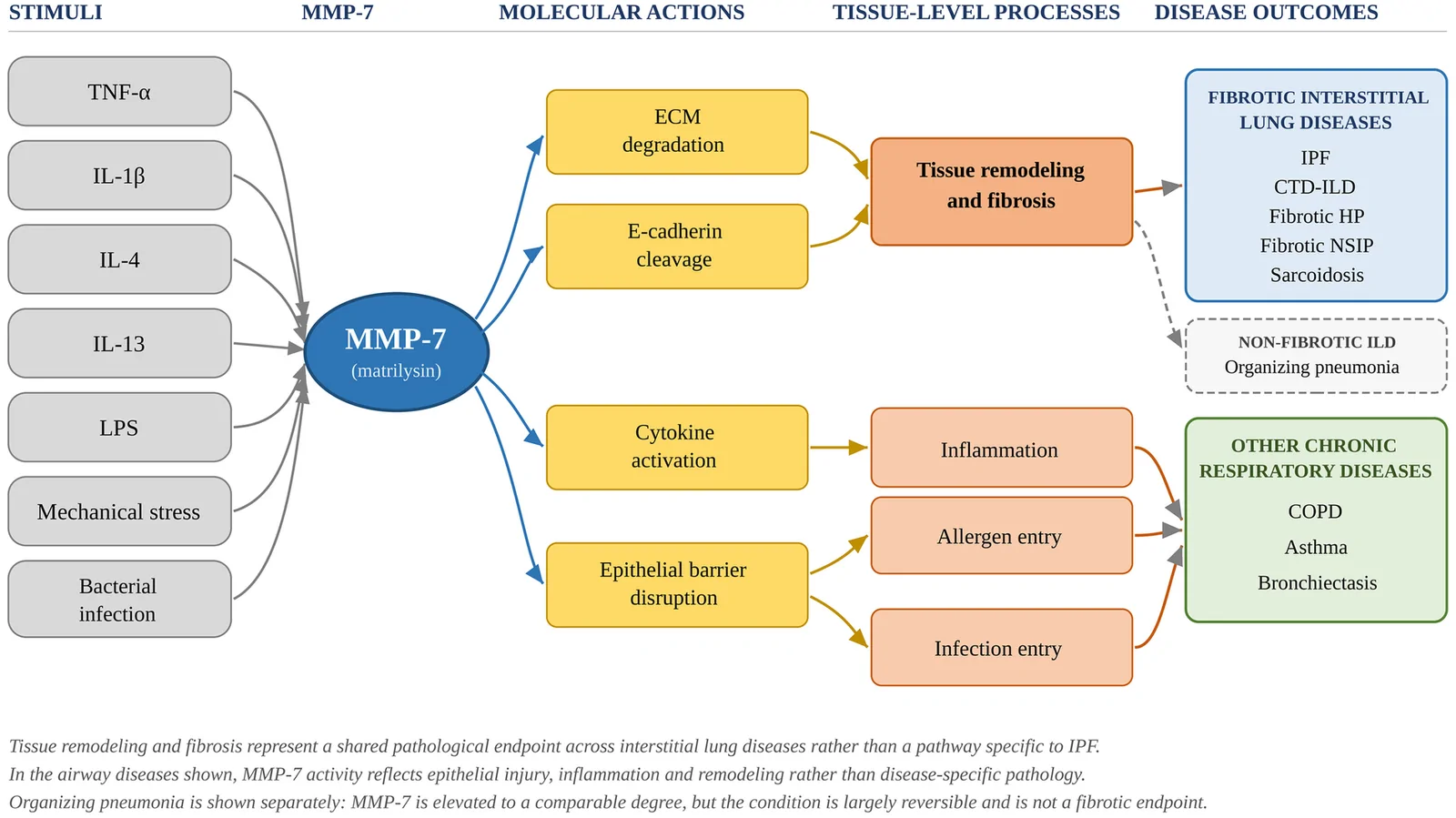
Expression and regulation of MMP-7 across chronic respiratory diseases. Tissue remodeling and fibrosis are shown as a shared pathological endpoint across interstitial lung diseases rather than as a pathway specific to IPF; in the airway diseases shown, MMP-7 activity reflects epithelial injury, inflammation and remodeling rather than disease-specific pathology. Organizing pneumonia is shown separately, outside the fibrotic endpoint, because MMP-7 is elevated to a comparable degree in a condition that is largely reversible. TNF-α, tumor necrosis factor-alpha; IL-1β, interleukin-1 beta; IL-4, interleukin-4; IL-13, interleukin-13; LPS, lipopolysaccharides; MMP-7, matrix metalloproteinase-7; ECM, extracellular matrix. E-cadherin, Epithelial cadherin; IPF, idiopathic pulmonary fibrosis; COPD, chronic obstructive pulmonary disease. Original figure created by the authors.

Regulation of MMP-7 activity does not, however, depend on expression alone. The enzyme is synthesized as pro-MMP-7 and requires proteolytic activation to acquire catalytic activity. Metalloproteinase activity is further controlled by endogenous tissue inhibitors of metalloproteinases (TIMPs), as well as by substrate availability and enzyme compartmentalization within the extracellular microenvironment (7–9).

Under pathological conditions characterized by persistent epithelial injury and chronic inflammation, dysregulation of these mechanisms can lead to sustained increases in MMP-7 expression and activity, contributing to disruption of the epithelial barrier and abnormal extracellular matrix remodeling (9, 16, 17).

### 3.4 Measurement of MMP-7: analytical considerations

Most clinical studies have quantified MMP-7 in peripheral blood, using either serum or plasma, although bronchoalveolar lavage fluid (BALF), induced sputum and lung tissue have also been examined in selected settings. Serum remains the most frequently analyzed specimen because of its accessibility, minimally invasive collection and suitability for longitudinal monitoring. Differences between serum and plasma measurements, together with variation in sample processing, storage conditions and freeze–thaw cycles, nevertheless contribute to inter-study variability and complicate direct comparison of absolute concentrations between cohorts.

Enzyme-linked immunosorbent assay (ELISA) is the most widely used method for measuring circulating MMP-7, owing to its simplicity, sensitivity and relatively low cost. Multiplex immunoassays have been employed increasingly, allowing simultaneous quantification of MMP-7 together with other biomarkers such as KL-6, CCL18, SP-D and periostin, and several of the studies discussed in this review used bead-based platforms of this kind (22). In research settings, immunohistochemistry has been used to assess tissue expression of MMP-7, particularly in alveolar and bronchial epithelial cells, while quantitative PCR has been applied to gene expression.

The consequences of this methodological heterogeneity are visible within the present review. The studies discussed in this review differ in analytical platform, antibody, calibration standard and reporting unit, and the cut-off values they propose differ correspondingly: a threshold of 3.91 ng/mL derived using bead-based multiplex assay (22) is not directly interchangeable with values obtained by ELISA in other cohorts, where reported median concentrations in IPF have been considerably higher (23). Thresholds also vary with the clinical question rather than the assay alone: the same group, using the same platform, proposed 3.91 ng/mL for distinguishing IPF from other ILDs (22) and 3.53 ng/mL for predicting PPF in non-IPF fibrotic ILD, with a sensitivity of 61% and a specificity of 74% (24). No internationally accepted reference range or standardized protocol currently exists. Harmonization of pre-analytical procedures, analytical methodology and clinically validated thresholds is therefore a prerequisite for the routine application of MMP-7 as a diagnostic and prognostic biomarker, and represents a more immediate obstacle to implementation than any remaining uncertainty about its biological relevance.

## 4 MMP-7 in interstitial lung diseases

### 4.1 MMP-7 in fibrotic ILD

The entities considered here are those in which fibrotic remodeling is present or may develop, and in which the available MMP-7 data relate principally to that fibrotic component. Sarcoidosis is included on this basis, since the evidence linking MMP-7 to sarcoidosis concerns chronic fibrotic disease rather than granulomatous inflammation, although non-fibrotic phenotypes predominate in unselected cohorts.

#### 4.1.1 MMP-7 in IPF

IPF is a chronic, progressive interstitial lung disease characterized by excessive extracellular matrix deposition and irreversible distortion of alveolar architecture. This fibrotic remodeling progressively impairs lung mechanics and gas exchange, frequently culminating in respiratory failure. Despite the availability of antifibrotic therapies that can slow disease progression, overall prognosis remains poor, with a median survival of approximately three to five years in the absence of lung transplantation. Early diagnosis remains challenging yet critically important for optimizing therapeutic outcomes. In this context, increasing attention has been directed toward circulating biomarkers, including matrix metalloproteinases such as MMP-7, as tools for earlier detection, disease stratification, and differentiation of IPF from other interstitial lung diseases. These advances underscore the need for more sensitive diagnostic approaches and the development of personalized management strategies (25–28).

Numerous studies have highlighted MMP-7 as a robust diagnostic and prognostic biomarker in IPF. Serum levels of MMP-7 are consistently elevated in patients with IPF compared with healthy controls and individuals with other interstitial or non-interstitial lung diseases, reflecting its involvement in active fibrotic remodeling of the lung parenchyma. Importantly, increased circulating MMP-7 concentrations correlate with disease severity, as demonstrated by declines in pulmonary function parameters such as forced vital capacity (FVC) and diffusing capacity for carbon monoxide (DLCO), as well as with radiological extent of fibrosis on HRCT. These associations support the potential utility of MMP-7 for disease characterization and risk stratification, while its role in longitudinal monitoring and therapeutic response assessment remains to be clinically validated. At the mechanistic level, MMP-7 contributes to epithelial barrier disruption through the proteolytic shedding of E-cadherin, impairing cell–cell adhesion. This has been proposed to facilitate epithelial–mesenchymal transition and fibrogenic remodeling, although the extent to which that pathway operates in human disease remains uncertain. This dual biological and clinical relevance supports the role of MMP-7 as a non-invasive biomarker in IPF (29–31).

Beyond its biomarker role, MMP-7 contributes directly to the pathogenesis of IPF. It is predominantly produced by alveolar type II epithelial cells in response to chronic injury, a population that exhibits aberrant activation and phenotypic plasticity in this disease, and the mechanisms involved are those described in Section 3.2 (32–34).

Beyond its diagnostic utility, MMP-7 has emerged as a prognostic biomarker in IPF, although the available evidence supports baseline measurement rather than serial monitoring. An individual participant data meta-analysis of nine studies and 1,664 patients with untreated IPF found that higher baseline MMP-7 was independently associated with mortality (adjusted hazard ratio 1.23, 95% CI 1.03–1.48) and with disease progression, defined as a 10% relative decline in FVC or death within 12 months (adjusted odds ratio 1.27, 95% CI 1.11–1.46), after adjustment for age, sex, smoking history and baseline FVC. In the same analysis, three-month change in MMP-7 was not associated with outcomes. Baseline concentrations may therefore assist in risk stratification and in identifying patients with a more aggressive fibrotic phenotype, whereas the value of serial measurement for tracking disease activity or treatment response remains unproven and requires prospective evaluation (24, 35–37).

#### 4.1.2 MMP-7 in CTD-ILD

CTD-ILD comprises a heterogeneous group of fibrosing lung disorders occurring in the context of systemic autoimmune diseases, most notably systemic sclerosis (SSc), rheumatoid arthritis (RA), and idiopathic inflammatory myopathies. ILD represents a major determinant of morbidity and mortality in these conditions, particularly in SSc, where pulmonary involvement is a leading cause of disease-related death. Early identification of CTD-ILD, prior to the development of irreversible fibrotic changes, is critical for timely therapeutic intervention and preservation of lung function. However, conventional diagnostic modalities, including HRCT and pulmonary function tests, often detect disease at relatively advanced stages. This limitation underscores the need for sensitive, non-invasive biomarkers capable of enabling earlier diagnosis and improving risk stratification in patients with CTD-ILD, with increasing interest in proteolytic enzymes such as MMP-7 as potential candidates (38–40).

Chen et al. compared serum matrix metalloproteinase concentrations in 80 patients with CTD-ILD, 100 patients with connective tissue disease (CTD) without pulmonary involvement, and 80 healthy controls, the CTD cohort comprising rheumatoid arthritis, systemic lupus erythematosus, polymyositis, Sjögren's syndrome, and systemic sclerosis. Serum MMP-3, MMP-7, and MMP-9 were all significantly higher in the CTD-ILD group than in either comparison group, but only MMP-7 correlated with markers of disease activity, showing positive associations with C-reactive protein (CRP), erythrocyte sedimentation rate, and rheumatoid factor, whereas MMP-2, MMP-3, and MMP-9 did not. Two caveats apply to this study: pulmonary function was assessed by spirometry alone, without measurement of diffusing capacity, and MMP-7 correlated positively rather than negatively with forced expiratory volume in 1 s (FEV1), FEV1/FVC, and FVC—a direction opposite to that reported in disease-specific cohorts and not addressed by the authors (5). In a large prospective cohort of patients with rheumatoid arthritis, Luedders et al. reported that individuals in the highest quartile of plasma MMP-7 levels had nearly a fourfold higher likelihood of prevalent ILD at baseline and more than a twofold increased risk of developing incident ILD during follow-up, compared with those with lower MMP-7 levels (41). Disease-specific cohorts provide more consistent functional data. In systemic sclerosis-associated ILD (SSc-ILD), each doubling of serum MMP-7 was associated with an adjusted decrease of 6.6 percentage points in FVC % predicted and 7.0 percentage points in DLCO % predicted, as well as with reduced transplant-free survival (4). At the molecular level, MMP-7 is released primarily by epithelial cells and macrophages in response to pro-inflammatory cytokines such as TNF-α and IL-1β, and contributes to extracellular matrix degradation and fibrotic remodeling; a further role in epithelial–mesenchymal transition has been proposed but remains contested. In line with these findings, elevated serum MMP-7 has been reported in both rheumatoid arthritis-associated ILD (RA-ILD) and SSc-ILD relative to patients with the same connective tissue disease but without ILD (3).

Histological data provide indirect support for this relationship, although the available evidence derives from IPF rather than CTD-ILD: in explanted IPF lungs, MMP-7 expression was increased in the more heavily fibrotic bases but not in the relatively spared apices, indicating that local MMP-7 abundance tracks the extent of established fibrosis (42). Whether a comparable relationship holds in CTD-ILD has not been directly demonstrated.

#### 4.1.3 MMP-7 in NSIP

NSIP occurs both as an idiopathic entity and, more commonly, secondary to connective tissue disease, in which it is the most frequent histological pattern, and is histologically subdivided into cellular and fibrotic patterns, the latter carrying a worse prognosis and a greater likelihood of progressive behavior. Distinguishing fibrotic NSIP from IPF is clinically important, since the two differ substantially in treatment and outcome yet may overlap radiologically.

Two studies have measured serum MMP-7 in NSIP populations, and both place it below IPF. Using Luminex xMAP in 139 patients—47 with IPF, 36 with non-IPF usual interstitial pneumonia (UIP), 14 with idiopathic NSIP, 29 with secondary NSIP and 13 with stage IV sarcoidosis—MMP-7 was significantly higher in IPF than in secondary NSIP (*p* < 0.001); the area under the curve for IPF vs. other ILD was 0.73, and a cut-off of 3.91 ng/mL yielded a sensitivity of 72.3% and a specificity of 66.3%, with a negative predictive value of 82.4% in that cohort (22). In a separate series using ELISA, median MMP-7 was 9.10 ng/mL in IPF compared with 6.50 ng/mL in NSIP (*p* = 0.007) and 4.35 ng/mL in healthy donors (23).

NSIP therefore occupies an intermediate position: MMP-7 is raised relative to healthy individuals but consistently lower than in IPF, consistent with the wider pattern that MMP-7 reflects the intensity of fibrotic remodeling rather than distinguishing diagnostic categories. Within that study, the negative predictive value may be the more informative parameter, since a low MMP-7 concentration argued against IPF whereas an elevated concentration lacked sufficient specificity to establish the diagnosis. This threshold and its performance are nevertheless specific to the population studied: predictive values depend on the prevalence of IPF in the population tested, and neither the cut-off nor the negative predictive value has been validated in independent cohorts. It should therefore not be applied as a general rule for excluding IPF. Notable gaps remain: no study has compared MMP-7 between cellular and fibrotic NSIP; the idiopathic NSIP subgroups examined have been small, and the difference between IPF and idiopathic NSIP did not reach significance in the larger of the two series; and the substantial difference in absolute concentrations between the studies reflects differing analytical platforms rather than differing biology. Data from mixed fibrotic ILD cohorts cannot be directly extrapolated to NSIP, and no study has specifically examined MMP-7 for prognostic assessment or longitudinal monitoring in this population. Their broader significance is considered below in the context of the progressive pulmonary fibrosis phenotype.

#### 4.1.4 MMP-7 in HP

HP is an immune-mediated interstitial lung disease caused by repeated inhalation of environmental antigens in susceptible individuals. Depending on disease duration and progression, HP may present as a predominantly inflammatory non-fibrotic phenotype or evolve into a chronic fibrotic form associated with irreversible architectural distortion, progressive respiratory impairment and poor outcomes. This clinical heterogeneity makes HP a particularly instructive setting in which to examine whether MMP-7 tracks fibrosis or diagnosis.

Most available data address the discrimination of IPF from HP rather than the characterization of HP itself. Plasma MMP-7 and MMP-1 concentrations are significantly higher in IPF than in subacute or chronic HP, and this contrast may have potential utility in the differential diagnosis of IPF and HP, although it does not establish MMP-7 as a diagnostic biomarker for HP (43). Within fibrotic non-IPF ILD populations that include fibrotic HP, higher baseline serum MMP-7 has been associated with subsequent development of PPF (24), indicating that MMP-7 carries prognostic information across fibrotic ILD irrespective of the underlying etiology.

It should be emphasized that direct comparisons of circulating MMP-7 between fibrotic and non-fibrotic HP are scarce, and the available evidence does not currently support the use of MMP-7 either to diagnose HP or to establish a fibrotic phenotype in an individual patient. Given that PPF may develop across multiple interstitial lung diseases, MMP-7 appears to reflect the fibrotic process rather than the underlying cause. Larger multicenter studies with explicit fibrotic and non-fibrotic strata are required before standardized thresholds can be proposed.

#### 4.1.5 MMP-7 in sarcoidosis

Sarcoidosis is a multisystem granulomatous disorder of unknown etiology characterized by non-caseating granulomas, most commonly affecting the lungs and intrathoracic lymph nodes. Although the disease is primarily driven by dysregulated immune responses rather than progressive fibrosis, a subset of patients develops chronic pulmonary sarcoidosis with irreversible fibrotic remodeling, impaired lung function and reduced quality of life.

Evidence regarding MMP-7 in sarcoidosis is divergent, and this divergence is itself informative. In one cohort, plasma MMP-7 was significantly higher in patients with sarcoidosis than in healthy controls; MMP-7 concentrations in both plasma and BALF were higher in patients with parenchymal infiltration than in those without lung lesions, and MMP-7 correlated negatively with pulmonary function, leading the authors to conclude that among the markers tested MMP-7 most closely reflected pulmonary disease status; these cross-sectional associations do not, however, establish prognostic value or clinical utility for longitudinal monitoring (44). By contrast, earlier work reported that plasma MMP-7 was not elevated in sarcoidosis relative to controls (43), and comparative studies have consistently found MMP-7 to be significantly lower in sarcoidosis than in IPF (22). Notably, transcriptomic profiling of sarcoidosis lung tissue has identified MMP-12 and ADAMDEC1, rather than MMP-7, as the most highly differentially expressed matrix-remodeling genes (45).

Taken together, these findings suggest that MMP-7 is more closely associated with fibrotic remodeling than with granulomatous inflammation *per se*, which may explain both its greater apparent relevance in patients with chronic fibrotic pulmonary sarcoidosis and its inconsistency across unselected sarcoidosis cohorts, in which non-fibrotic phenotypes predominate. Discrepancies between studies also reflect differences in disease stage, patient selection, specimen type and assay methodology. Unlike IPF, where MMP-7 has demonstrated reproducible diagnostic and prognostic value, its clinical utility in sarcoidosis is not established, and larger prospective studies stratified by fibrotic phenotype are required.

#### 4.1.6 MMP-7 in PPF

Taken together, the findings across the fibrotic entities considered above suggest that MMP-7 may be more closely related to fibrotic disease behavior than to a specific ILD diagnosis. PPF is not confined to IPF but may develop across a range of fibrosing ILDs. The 2022 ATS/ERS/JRS/ALAT guideline defines PPF as a disease behavior rather than a specific diagnosis, requiring at least two of three features within one year: worsening symptoms, physiological decline (≥5% absolute FVC or ≥10% absolute DLCO predicted), and radiological progression, without an alternative explanation (46).

Direct evidence supports this concept. In a cohort of 79 patients with fibrotic non-IPF ILD, comprising CTD-ILD, fibrotic HP, sarcoidosis and idiopathic NSIP, serum MMP-7 was significantly higher in patients who subsequently developed PPF than in those who did not (3.85 vs. 2.41 ng/mL). MMP-7 remained independently associated with PPF after adjustment for sex, age and smoking history (adjusted OR, 1.26; 95% CI, 1.03–1.55), whereas MMP-1 did not differ between groups (24). The association of MMP-7 with PPF in a mixed-etiology cohort suggests that circulating MMP-7 may reflect biological features of progressive fibrotic disease shared across different ILD diagnoses. These findings support risk stratification rather than establishing MMP-7 as a diagnostic or monitoring biomarker for PPF, and external validation in larger, disease-stratified cohorts is required.

### 4.2 MMP-7 in non-fibrotic ILD

#### 4.2.1 MMP-7 in OP

Organizing pneumonia, including cryptogenic organizing pneumonia (COP), is characterized by intra-alveolar granulation tissue plugs and variable interstitial inflammation. In contrast to fibrotic interstitial lung diseases, OP is generally considered reversible and responds well to corticosteroid therapy. Nevertheless, extracellular matrix remodeling is integral to the reparative process, suggesting a potential role for MMP-7.

Clinical data are limited but conceptually important. In a comparative study of BALF and lung tissue, MMP-7 levels were higher in both COP and UIP than in normal controls, but did not differ significantly between COP and UIP, and the pattern and degree of MMP-7 expression in lung tissue were similar in the two conditions (47). Patients with COP have likewise been included within non-IPF ILD comparator groups used to develop multimarker diagnostic indices incorporating MMP-7 (48).

This observation carries weight beyond OP itself. That a corticosteroid-responsive, largely reversible condition shows MMP-7 elevation comparable to that of established fibrotic disease argues against interpreting circulating or airway MMP-7 as a specific marker of irreversible fibrosis, and supports the broader conclusion of this review that MMP-7 reflects active epithelial injury and matrix turnover rather than a fixed fibrotic burden.

## 5 MMP-7 in other chronic respiratory diseases

### 5.1 MMP-7 in obstructive airway diseases

#### 5.1.1 MMP-7 in COPD

COPD is a progressive respiratory disorder characterized by persistent and incompletely reversible airflow limitation, driven principally by chronic exposure to noxious particles and gases. The resulting inflammation promotes extracellular matrix degradation, protease–antiprotease imbalance, and structural remodeling of the lung, with emphysematous destruction of alveolar walls and narrowing of small airways that impair gas exchange. Matrix metalloproteinases, including MMP-7, have been implicated in these processes (6, 49, 50).

Additional risk factors, including metabolic syndrome, lifestyle and environmental exposures, are increasingly recognized as contributors to COPD pathogenesis and clinical course (51, 52). Within this context, MMP-7 has attracted attention because of its role in extracellular matrix breakdown, a central process in tissue destruction and airway remodeling (53–55).

Several studies have reported elevated MMP-7 levels in serum and BALF from patients with COPD compared with healthy controls, although these findings are not uniform. Proteomic analyses have identified MMP-7 among biomarkers associated with accelerated FEV1 decline in smoking-related COPD (56). Montaño et al. found that higher MMP-7 levels were associated with lower FEV1 in patients with COPD exposed to biomass smoke, suggesting that sustained MMP-7 activity may contribute to airway remodeling and airflow limitation (57). MMP-7 also contributes to matrix remodeling and inflammatory signaling in chronic airway disease (58). Increased metalloproteinase activity has likewise been reported in BALF, bronchial tissue, and the systemic circulation (59). Taken together, these findings suggest that MMP-7 may reflect structural lung injury and ongoing remodeling in COPD, although not consistently across cohorts.

MMP-7 participates in airway inflammation through mechanisms that have been defined largely in experimental lung injury rather than in COPD itself. In murine acute lung injury, MMP-7 sheds syndecan-1–chemokine complexes from the epithelial surface, generating the transepithelial gradient that directs neutrophil influx into the alveolar space; in MMP-7-deficient animals, neutrophils remain confined to the pulmonary interstitium (60). MMP-7 also mediates E-cadherin ectodomain shedding in injured lung epithelium, loosening intercellular junctions during repair (15), and pro-inflammatory effects have additionally been demonstrated in non-pulmonary models of acute inflammation (61). Whether these mechanisms operate in the chronically injured COPD airway has not been directly established, and MMP-7 is therefore best regarded as a plausible rather than a demonstrated contributor to persistent inflammatory activity in this disease.

Although the evidence remains limited, persistently elevated MMP-7 in COPD may reflect enhanced matrix turnover and accelerated lung function decline, and inverse correlations between MMP-7 and FEV1 have been reported (57). Exacerbations are characterized by intensified extracellular matrix remodeling and inflammatory activation involving metalloproteinases (62). MMP-7 has therefore been proposed as a potential biomarker of disease progression and risk stratification in COPD, although its value for longitudinal monitoring and prediction of clinical outcomes remains insufficiently validated (6). Sustained MMP-7 activity may perpetuate epithelial injury and aberrant tissue repair, contributing to progressive structural remodeling of the lung and subsequent functional decline.

#### 5.1.2 MMP-7 in asthma

Bronchial asthma is a heterogeneous chronic inflammatory airway disease characterized by variable airflow limitation, bronchial hyperresponsiveness, and progressive airway remodeling. Severe asthma represents a phenotype associated with persistent inflammation, remodeling, and reduced therapeutic responsiveness (63). This heterogeneity has driven interest in molecular biomarkers capable of improving phenotypic and endotypic characterization (64, 65).

Preliminary evidence suggests that MMP-7 expression is increased within the airway epithelium in asthma, and experimental studies indicate that epithelial-derived MMP-7 participates in the orchestration of allergic airway inflammation (66). Airway remodeling, a hallmark of chronic asthma, involves epithelial dysfunction, extracellular matrix remodeling, and smooth muscle alterations (67, 68). Given its proteolytic activity and role in epithelial repair, MMP-7 may participate in the crosstalk between epithelial injury, immune activation, and tissue remodeling (66, 67, 69). Eosinophilic inflammation may further contribute to the cytokine-rich microenvironment that promotes MMP-7 expression (70). MMP-7 may therefore represent a molecular link between epithelial dysfunction, inflammation, and airway remodeling in asthma (66, 69).

These effects appear to operate through several interconnected mechanisms. As a protease regulating extracellular matrix turnover and processing bioactive mediators, MMP-7 participates in epithelial homeostasis and in the orchestration of immune responses (66, 71). Disruption of the airway epithelial barrier, a recognized feature of asthma, facilitates allergen penetration and sustains immune activation (72). Mediators such as TNF-α further compromise epithelial integrity (73). Epithelial-derived pathways are also increasingly recognized as drivers of airway fibrosis, in which MMP-7 may participate through epithelial–mesenchymal crosstalk (66, 74). Through these combined actions MMP-7 may link barrier dysfunction, chronic inflammation, and the structural changes that characterize severe asthma.

The growing emphasis on asthma phenotyping has intensified the search for biomarkers capable of capturing underlying inflammatory and remodeling processes. Given its involvement in epithelial dysfunction and tissue remodeling, MMP-7 has emerged as a candidate that may complement existing inflammatory markers, although current evidence remains insufficient to support routine clinical use (75–78).

### 5.2 MMP-7 in suppurative airway diseases

#### 5.2.1 MMP-7 in bronchiectasis

Bronchiectasis is a chronic suppurative airway disease characterized by irreversible bronchial dilatation, persistent airway inflammation, and recurrent respiratory infection. Etiologies include prior respiratory infection, autoimmune and connective tissue disease, immunodeficiency, genetic disorders such as cystic fibrosis and primary ciliary dyskinesia, and airway obstruction (79–81). Regardless of origin, a self-perpetuating cycle of impaired airway clearance, microbial colonization, chronic inflammation and progressive structural damage sustains disease progression (79, 81, 82).

MMP-7 has been examined as part of broader protease profiling in bronchiectasis rather than as a biomarker in its own right. In induced sputum from 86 patients and 8 healthy controls, MMP-1, -3, -7, -8 and -9 were all elevated relative to controls, although the difference for MMP-7 was of borderline significance compared with the other metalloproteinases, and the reported associations with airway microbiota composition involved MMP-2 and MMP-8 (83). MMP-2, MMP-8 and MMP-9 remain the proteases most consistently linked to airway remodeling and disease severity in this population (84), and evidence establishing MMP-7 as an independent diagnostic or prognostic biomarker is correspondingly limited.

During chronic infection, airway epithelial and immune cells upregulate MMP-7 in response to inflammatory and microbial stimuli, potentially contributing to extracellular matrix degradation, inflammatory signaling, and tissue remodeling that sustain the cycle of infection and injury characteristic of bronchiectatic lung disease (85).

Protease-mediated tissue remodeling appears to contribute to disease progression, particularly in patients with chronic bacterial colonization. Persistent infection with *Pseudomonas aeruginosa* is associated with heightened airway inflammation and worse clinical outcomes (86, 87). Experimental work indicates that *P. aeruginosa* infection modulates matrix metalloproteinase expression and induces distinct transcriptional responses in airway epithelial cells (88). Taken together, these observations indicate that chronic bacterial infection modulates airway metalloproteinase expression, although the specific contribution of MMP-7 to tissue injury and disease severity in bronchiectasis has not been established (86–88).

Prospective multicenter studies are required to establish whether MMP-7 has diagnostic, prognostic or monitoring value in bronchiectasis before any clinical role can be considered.

## 6 Discussion

MMP-7 has shown promise as a circulating biomarker in chronic respiratory disease, most clearly in fibrosing ILD. Elevated concentrations have been reported across a range of conditions, but the strength and consistency of that signal vary considerably: reproducible in IPF and CTD-ILD, divergent in sarcoidosis, non-uniform in COPD, and largely experimental or of borderline significance in asthma and bronchiectasis. Where elevation is observed, it appears to track epithelial injury and extracellular matrix turnover rather than any single disease process, which is what makes MMP-7 informative about disease activity while limiting its value as a diagnostic label (3, 4, 36, 37, 89).

Among all respiratory disorders, the strongest evidence currently supports the clinical utility of MMP-7 in progressive fibrosing interstitial lung diseases rather than in ILDs as a whole, and particularly in IPF and CTD-ILD. In IPF, MMP-7 has demonstrated robust diagnostic performance and reproducible associations with disease severity, pulmonary function decline, disease progression, and mortality. The consistency of these findings across multiple cohorts suggests that MMP-7 is one of the most clinically relevant non-invasive biomarkers currently available in fibrotic lung disease. This may reflect the central role of epithelial injury and progressive extracellular matrix remodeling in IPF pathogenesis, processes in which MMP-7 appears closely linked to disease-driving mechanisms (22, 24, 43, 90, 91).

From a clinical perspective, MMP-7 should be interpreted according to disease context rather than as a standalone diagnostic marker. Current evidence indicates that the magnitude and clinical significance of MMP-7 elevation vary across chronic respiratory diseases. The strongest evidence is available in fibrosing interstitial lung diseases, particularly IPF and CTD-ILD, where elevated serum concentrations are consistently associated with disease presence, severity, progression and mortality. In contrast, although MMP-7 is frequently increased in COPD, severe asthma, bronchiectasis, sarcoidosis, HP and OP, its elevation primarily reflects ongoing epithelial injury and tissue remodeling rather than disease-specific pathology. MMP-7 therefore appears most useful as a diagnostic and prognostic biomarker in fibrosing ILDs, whereas in other chronic respiratory diseases the available data do not yet define a clinical role. Evidence for serial measurement is limited in all of these conditions, and the utility of MMP-7 for monitoring disease activity or treatment response should be regarded as unestablished. Importantly, no universally accepted disease-specific cut-off values or reference ranges have been established, and MMP-7 should be interpreted in conjunction with clinical findings, imaging, pulmonary function testing and, wherever possible, complementary biomarkers.

The greater clinical utility of MMP-7 in fibrosing interstitial lung diseases likely reflects the central role of epithelial injury and aberrant extracellular matrix remodeling in the pathogenesis of these disorders. In IPF and other progressive fibrosing ILDs, MMP-7 has been implicated in epithelial–mesenchymal transition, myofibroblast activation and collagen deposition, although, as noted in Section 3.2, the contribution of these processes to human pulmonary fibrogenesis remains incompletely defined; circulating levels nevertheless track disease severity, pulmonary function decline and clinical progression. In contrast, COPD, asthma and bronchiectasis are characterized by more heterogeneous inflammatory mechanisms in which extracellular matrix remodeling represents only one component of disease pathobiology. MMP-7 elevations in these conditions are therefore less disease-specific and provide more limited diagnostic and prognostic information (Table 1).

**Table 1:** Evidence for MMP-7 as a biomarker across chronic respiratory diseases: key findings, supported clinical roles and principal limitations.

Disease	Key evidence	Supported clinical role	**Main limitation**
Fibrotic interstitial lung diseases			
IPF	Elevated in serum, plasma, BALF and lung tissue relative to healthy controls and other ILDs, and inversely correlated with FVC and DLCO and with the radiological extent of fibrosis (29–31). Baseline concentrations predict mortality (adjusted HR 1.23, 95% CI 1.03–1.48) and disease progression (adjusted OR 1.27, 95% CI 1.11–1.46) in an individual participant data meta-analysis (35–37).	Potential diagnostic and prognostic role; limited evidence for monitoring; no established role in screening.	Three-month change in MMP-7 is not associated with outcomes (31, 35). The 3.91 ng/mL cut-off proposed for distinguishing IPF from other ILDs (22) is study-specific and lacks external validation; assay and population heterogeneity limit generalizability.
CTD-ILD (SSc-ILD, RA-ILD)	Elevated relative to connective tissue disease without lung involvement and to healthy controls, and associated with prevalent and incident ILD in rheumatoid arthritis (3, 5, 38, 41). In SSc-ILD, higher concentrations correlate with reduced FVC and DLCO and with shorter transplant-free survival (4, 41).	Potential diagnostic and prognostic role; limited evidence for screening (41); no established role in monitoring.	Limited number of cohorts, heterogeneous CTD-ILD populations and disease subtypes, lack of standardized thresholds, and screening evidence based on a single prospective cohort; no serial measurements.
Fibrotic NSIP	Serum concentrations are intermediate between healthy controls and IPF (median 6.50 vs. 9.10 ng/mL) and significantly lower than in IPF in secondary NSIP. A study-specific cut-off of 3.91 ng/mL was proposed for distinguishing IPF from other ILDs (22, 23).	Potential role in differential diagnosis from IPF; no established prognostic, monitoring or screening role.	The 3.91 ng/mL cut-off has not been externally validated, and its reported negative predictive value of 82.4% depends on the prevalence and case mix of the source cohort.
Fibrotic HP	Concentrations are lower than in IPF (43), and were elevated in a single fibrotic non-IPF ILD cohort in patients who subsequently developed PPF (24).	Potential role in distinguishing IPF from HP; insufficient evidence for diagnostic, prognostic or monitoring use in HP itself.	Small and heterogeneous cohorts; no direct comparison between fibrotic and non-fibrotic HP; no validated thresholds and no serial data.
Sarcoidosis	Findings are conflicting: concentrations are consistently lower than in IPF (22, 43, 44), and were elevated relative to controls and higher with parenchymal infiltration in one cohort but not elevated in others (44).	Limited and inconsistent evidence for diagnostic or disease-phenotyping applications; no established role in prognosis, monitoring or screening.	Conflicting evidence across cohorts; associations are cross-sectional; findings differ by disease stage, specimen and assay, and no validated thresholds exist.
Non-fibrotic interstitial lung diseases			
Organizing pneumonia	Elevated in BALF and lung tissue relative to controls but not significantly different from usual interstitial pneumonia, despite the largely reversible clinical course of the disease, suggesting that MMP-7 elevation is not specific to irreversible fibrotic remodeling (47, 48).	No established clinical role.	Very limited clinical evidence; MMP-7 does not distinguish organizing pneumonia from fibrotic disease, and its elevation should not be interpreted as evidence of an equivalent fibrotic trajectory.
Obstructive airway diseases			
COPD	Inversely correlated with FEV1 and associated with accelerated FEV1 decline in proteomic analyses (6, 56, 57), although several cohorts report no elevation relative to controls.	Preliminary prognostic potential for disease severity and progression; no established diagnostic, monitoring or screening role.	Inconsistent findings across studies, with substantial biological and methodological heterogeneity; no serial measurements.
Asthma	Epithelial expression is increased in the airway, and experimental models implicate MMP-7 in allergic airway inflammation and remodeling (66, 67, 69).	No established clinical role; potential mechanistic relevance.	Evidence is predominantly experimental, with insufficient clinical validation.
Suppurative airway diseases			
Bronchiectasis	Matrix metalloproteinases are elevated in airway secretions, but the difference for MMP-7 was of borderline significance compared with the other metalloproteinases measured, and the reported associations with lung function and disease severity involved MMP-2, MMP-8 and MMP-9 (83, 84).	No established diagnostic, prognostic, monitoring or screening role for MMP-7.	Reported clinical associations concern other metalloproteinases rather than MMP-7; sparse evidence and no validated thresholds.

BALF, bronchoalveolar lavage fluid; COPD, chronic obstructive pulmonary disease; CTD-ILD, connective tissue disease-associated interstitial lung disease; DLCO, diffusing capacity of the lung for carbon monoxide; FEV1, forced expiratory volume in 1 s; FVC, forced vital capacity; HP, hypersensitivity pneumonitis; ILD, interstitial lung disease; IPF, idiopathic pulmonary fibrosis; MMP, matrix metalloproteinase; NSIP, nonspecific interstitial pneumonia; PPF, progressive pulmonary fibrosis; RA-ILD, rheumatoid arthritis-associated interstitial lung disease; SSc-ILD, systemic sclerosis-associated interstitial lung disease; UIP, usual interstitial pneumonia. The supported clinical role column summarizes the applications suggested by the available evidence and reflects the current state of that evidence rather than established clinical utility. “Potential” or “preliminary” roles indicate suggestive evidence that remains insufficiently validated for routine clinical use, whereas “no established role” indicates that the available evidence does not currently support a clinical application. Each application was judged against a specific requirement: diagnosis required comparison with a relevant control or comparator group; prognostication required baseline measurement linked to a subsequent clinical outcome; monitoring required serial measurement related to change in disease activity or treatment response, a design rarely reported for MMP-7; and screening required measurement in a population at risk but without established disease. The main limitation column summarizes the principal factors limiting interpretation or clinical translation, including small or heterogeneous cohorts, conflicting findings, absent standardized thresholds, lack of external validation, and limited longitudinal evidence. Molecular and experimental studies were treated as supportive biological evidence but were not used on their own to establish a clinical role. None of the applications listed is validated for routine clinical practice.

Although MMP-7 performs well in fibrosing ILDs, it is unlikely to replace existing biomarkers as a standalone test. KL-6 is among the most extensively validated markers of alveolar epithelial injury and has shown high sensitivity for detecting ILD; SP-D likewise reflects alveolar epithelial damage and surfactant dysfunction, whereas CCL18 is more closely associated with macrophage activation and fibrogenesis. In a study that compared MMP-7, CCL18, KL-6, SP-A and SP-D directly within a single IPF cohort, MMP-7, KL-6 and SP-D were significantly elevated relative to healthy controls and to patients with bacterial pneumonia, and multivariate survival analysis identified MMP-7 and KL-6 as independent predictors of outcome (92). These markers therefore appear complementary rather than competing, MMP-7 contributing information about extracellular matrix remodeling that the pneumoproteins do not capture. It should be acknowledged, however, that MMP-7 does not outperform KL-6 in every setting: in a cohort with Sjögren's syndrome-associated ILD, KL-6 predicted survival whereas MMP-7 did not differ between survivors and non-survivors (93). Growing evidence accordingly favors multimarker panels incorporating MMP-7 together with KL-6, SP-D and CCL18 over reliance on any single biomarker.

The central insight from the current literature is that MMP-7 lacks sufficient disease specificity to serve as an independent biomarker. Pulmonary fibrosis is not unique to IPF but represents a shared endpoint of several interstitial lung diseases, and elevated MMP-7 is best understood as marking the presence and activity of that shared process rather than any single diagnostic label. Consistent with this, raised concentrations have been reported in fibrotic HP, CTD-ILD and chronic fibrotic sarcoidosis, and higher baseline levels predict subsequent PPF in fibrotic non-IPF ILD (24). The practical consequence is that MMP-7 is most likely to be useful within multimarker strategies combining it with complementary markers such as KL-6, SP-D and CCL18, rather than in isolation (92, 94, 95).

Two important considerations should temper this interpretation. First, MMP-7 elevation is not confined to irreversible fibrosis: comparable levels have been documented in organizing pneumonia, a largely reversible condition (47), indicating that MMP-7 more plausibly indexes active epithelial injury and matrix turnover than accumulated fibrotic burden. Second, because this is a narrative review rather than a systematic review or meta-analysis with access to individual patient data, no quantitative comparison between fibrotic and non-fibrotic states was undertaken; most primary studies do not stratify their cohorts in this way. Prospective studies designed with explicit fibrotic and non-fibrotic strata therefore represent a priority for the field, and are discussed further below. Subject to these caveats, the clinical utility of MMP-7 appears more closely related to the presence and activity of fibrotic remodeling than to the underlying etiology of the disease.

Although MMP-7 is an appealing therapeutic target because of its involvement in extracellular matrix remodeling, epithelial injury, and fibrotic processes, therapeutic strategies aimed at inhibiting this enzyme have not yet translated into clinical practice. Experience with matrix metalloproteinase inhibitors has highlighted the substantial biological complexity of this approach. Early broad-spectrum inhibitors, developed primarily in oncology, failed to demonstrate sufficient efficacy in phase III trials and were associated with dose-limiting musculoskeletal toxicity, an adverse effect attributed mainly to concomitant inhibition of ADAM-10 and ADAM-17 rather than to inhibition of the targeted metalloproteinases themselves (96). The subsequent development of more selective inhibitors did not fully overcome these limitations, consistent with the recognition that individual metalloproteinases may exert both pathogenic and protective functions. Consequently, indiscriminate inhibition may interfere with physiological tissue homeostasis and repair and may ultimately prove counterproductive.

These considerations are particularly relevant to MMP-7, which participates not only in pathological remodeling but also in physiological epithelial repair, mucosal antimicrobial defense, and regulated neutrophil transmigration. Broad systemic inhibition could therefore interfere with essential host-protective mechanisms. Accordingly, current evidence supports a considerably more established role for MMP-7 as a diagnostic and prognostic biomarker than as a therapeutic target. Nevertheless, experimental data provide a rationale for continued investigation: in a rheumatoid arthritis-associated ILD model, MMP-7 inhibition using small interfering RNA or a pharmacological inhibitor prevented collagen-I synthesis in pleural mesothelial cells and attenuated adjuvant-induced subpleural inflammation and fibrosis *in vivo* (97). Selective MMP-7 inhibition, monoclonal antibodies and RNA-based strategies may thus offer future opportunities to modulate pathological activity while preserving physiological function, although no such approach has yet entered clinical evaluation in fibrosing lung disease.

Several limitations currently hinder the routine clinical implementation of MMP-7. Available studies differ substantially in patient selection, disease stage, biological sample type, assay methodology, and reported cut-off values. Moreover, many cohorts are relatively small, retrospective, and single-center, limiting the generalizability of their findings. Standardization of analytical methods, establishment of clinically meaningful reference thresholds, and validation in large prospective multicenter studies are therefore essential before MMP-7 can be incorporated into routine respiratory practice. A further limitation is the lack of disease specificity: elevated MMP-7 concentrations have been reported across several chronic respiratory diseases, and a raised value therefore cannot be interpreted in isolation.

## 7 Future research prospects

Future investigations should focus on the prospective clinical validation of MMP-7 across a broad spectrum of chronic respiratory diseases. Particular attention should be given to progressive fibrosing interstitial lung diseases, where the most consistent evidence currently supports its association with disease severity and progression, while further studies are required to clarify its clinical role in COPD, asthma, bronchiectasis, sarcoidosis, HP and OP. Prospective cohorts with clearly defined fibrotic and non-fibrotic phenotypes should determine whether MMP-7 can distinguish patients with stable disease from those who subsequently develop PPF, whether it can separate slowly from rapidly progressive disease—a question the available data do not address—and whether serial measurements add prognostic information beyond established clinical, physiological and radiological measures.

Equally important is the standardization of analytical methodology. Harmonized protocols for sample collection, processing, storage and quantification across biological matrices—including serum, plasma, sputum and BALF—are needed. Analytical platforms such as ELISA and multiplex immunoassays should undergo rigorous analytical and clinical validation, with particular attention to calibration, reporting units and pre-analytical variables. Such standardization is essential for improving reproducibility, facilitating comparisons between studies, and establishing clinically meaningful reference ranges and validated diagnostic or prognostic thresholds.

Another promising area is the investigation of MMP-7 following acute lung injury. Persistent epithelial injury, inflammation, microvascular dysfunction and fibroblast activation may contribute to fibrotic remodeling after moderate-to-severe COVID-19 pneumonia (98). In a post-COVID cohort, serum MMP-7 was elevated both during acute infection and one year after recovery, and higher levels were associated with persistent HRCT abnormalities and impaired gas exchange (99). These findings indicate an association with residual pulmonary abnormalities rather than demonstrating ongoing fibrogenic activity; prospective longitudinal studies are required to determine whether post-injury MMP-7 levels identify patients at risk of developing established pulmonary fibrosis.

Multimarker approaches also warrant further investigation. Combining MMP-7 with complementary biomarkers, including KL-6, CCL18, periostin and inflammatory mediators, may improve diagnostic discrimination and risk stratification compared with single-marker approaches. Prospective models integrating MMP-7 with HRCT features, pulmonary function parameters, clinical phenotype and serial biomarker measurements would be particularly informative, and would support earlier recognition of progressive disease and more individualized monitoring.

Finally, the therapeutic implications of MMP-7 biology remain experimental. Further preclinical studies are required to determine whether selective modulation of MMP-7 can attenuate pathological tissue remodeling while preserving its physiological functions. Given the limitations of non-selective metalloproteinase inhibition discussed above, therapeutic targeting of MMP-7 should currently be regarded as a hypothesis-generating avenue rather than an established translational strategy.

## 8 Conclusions

MMP-7 is a promising non-invasive biomarker associated with epithelial injury, extracellular matrix remodeling, and aberrant tissue repair across chronic respiratory diseases. Rather than being disease-specific, its elevation appears to reflect shared pathological processes, with the strongest evidence currently supporting its relevance in fibrosing interstitial lung diseases, particularly IPF and CTD-ILD.

In these conditions, increased circulating MMP-7 levels are associated with disease severity, functional impairment, disease progression, and adverse outcomes. Higher baseline MMP-7 has also been associated with the subsequent development of progressive pulmonary fibrosis in fibrotic non-IPF ILD cohorts, although whether it can identify particularly rapid progressors remains untested. In COPD, asthma, and bronchiectasis, MMP-7 may reflect airway injury, inflammation, and tissue remodeling, but its clinical utility remains less well established.

The routine clinical implementation of MMP-7 is currently limited by heterogeneity in patient populations, biological specimens, analytical methods, and reported thresholds, as well as by the predominance of small and retrospective studies. Standardized assays and large prospective multicenter validation studies are therefore needed.

Integration of MMP-7 with complementary biomarkers, imaging, pulmonary function parameters, and longitudinal clinical data may ultimately improve early diagnosis, risk stratification, recognition of progressive pulmonary fibrosis and individualized management, particularly in patients with progressive fibrotic lung disease.
